# Draft genome sequence of *Rhodococcus pyridinivorans* IEGM 66, a betulin biotransformer

**DOI:** 10.1128/mra.00687-25

**Published:** 2025-08-20

**Authors:** Natalia A. Plotnitskaya, Anastasiia V. Krivoruchko, Irina B. Ivshina

**Affiliations:** 1 Perm Federal Research Center of the Ural Branch of the Russian Academy of Sciences 307499, Perm, Russia; 2 Perm State University 64949, Perm, Russia; DOE Joint Genome Institute, Berkeley, California, USA

**Keywords:** *Rhodococcus rhodochrous*, betulin, biotransformation

## Abstract

We report the draft genome sequence of *Rhodococcus pyridinivorans* IEGM 66. This strain is able to transform a pentacyclic triterpenoid, betulin, commonly used as a scaffold for synthesizing bioactive compounds. Using the obtained sequence, putative genes encoding enzymes involved in the oxidative transformation of betulin were revealed.

## ANNOUNCEMENT

Oxidized derivatives of betulin, a lupane-type pentacyclic triterpenoid of plant origin, exhibit pronounced antimicrobial, antitumor, anti-inflammatory, and other activities (1–3). Recent efforts have focused on the biological transformation of triterpenoids using microbial enzymatic activity. Biocatalysis enables the production of target compounds with high regio- and stereoselectivity in a single technological step, under mild conditions (normal temperature and pressure), in a nonaggressive reaction medium, and with minimal environmental impact (4, 5).

Among the microorganisms widely used in biotechnology, actinomycetes of the genus *Rhodococcus* are particularly notable (6). This group of microorganisms has been shown to catalyze oxidative transformations of organic compounds across nearly all known classes (7–9). The *R. pyridinivorans* strain IEGM 66 (formerly *R. rhodochrous*), isolated from oil-polluted soil in 1980 using the enrichment culture method, is deposited in the Regional Specialized Collection of Alkanotrophic Microorganisms (acronym IEGM, WFCC number 285, http://www.iegmcol.ru/strains/rhodoc/pyridin/r_pyridin66.html). This strain can transform up to 60% of betulin at initial concentrations of 1–3 g/L in a mineral salt medium within 24 h (10, 11). The main derivative, identified as 3-oxo-betulin (betulone), demonstrates antitumor activity and serves as a promising precursor for synthesizing pharmacologically significant compounds (12, 13).

DNA was isolated from five-year-old lyophilized *R. pyridinivorans* IEGM 66 cells grown in Luria-Bertani (LB) broth at 28°C and 160 rpm for 28 h using the MagMAX DNA Multi-Sample Ultra 2.0 Kit (Thermo Fisher Scientific) and a KingFisher Flex automatic station (Thermo Fisher Scientific), following the manufacturer’s protocol. The quantity of DNA after isolation was >500 ng. The 100 ng was used for sequencing. A paired-end library (2 × 100 nucleotides [nt]) was produced using Illumina DNA Prep and sequenced on an Illumina NovaSeq 6000 instrument. A total number of 23,772,942 reads was obtained. Demultiplexing of the sequenced reads was performed with Illumina bcl2fastq (version 2.20). Adapters were trimmed with Skewer (version 0.2.2) (14). The quality of the FASTQ files was analyzed with FastQC (version 0.11.5-cegat) (15). The data were assembled with SPAdes v.3.14.1 (16) at the scaffold level. Assembly statistics were computed using QUAST 5.2.0 (17). The assembly consisted of 99 contigs with a total sequence length of 4,948,207  bp, an N50 value of 109,782  bp, a GC content of 68%, and coverage of 482×. The annotation of coding sequences (CDS) was performed using PGAP 5.3 (annotation method “best-placed reference protein set,” GeneMarkS-2+) (18) and RAST 2.0 (annotation scheme RASTtk) (19). Default parameters were used for all software unless otherwise specified.

For taxonomic classification, average nucleotide identity (ANI) was calculated using the online tool https://www.ezbiocloud.net/tools/ani (20), and the highest OrthoANIu values of 94.87% and 98.91% were obtained for *R. rhodochrous* (NCBI: txid1829) and *R. pyridinivorans* (NCBI: txid103816) species, respectively. Digital DNA–DNA hybridization (dDDH) was calculated using GGDC 4.0 (Genome-to-Genome Distance Calculator 4.0, https://ggdc.dsmz.de/ggdc.php) (21, 22). The calculated dDDH values of 58.6%–59.2% with *R. rhodochrous* and 89.3%–90.4% with *R. pyridinivorans* confirmed the taxonomic classification of strain IEGM 66 as *R. pyridinivorans*.

A total of 4,592 coding sequenses (CDSs), 4,450 CDSs with protein, 3 rRNAs (5S, 16S, and 23S), and 54 tRNAs were found in the *R. pyridinivorans* IEGM 66 genome. Among CDSs, 45 CDSs for monooxygenases, 23 for dioxygenases, 10 for hydroxylases, 7 for peroxidases, and 256 for dehydrogenases were identified. Among the oxygenases, genes encoding the following enzymes were detected: 10 CYP450s, 7 cyclohexanone monooxygenases, 2 3-ketosteroid-9α-monooxygenases, 2 steroid C27-monooxygenases, 1 catechol 1,2-dioxygenase (EC 1.13.11.1), 1 2,3-dihydroxybiphenyl 1,2-dioxygenase (EC 1.13.11.39), 2 benzoate 1,2-dioxygenases (EC 1.14.12.10), 1 homogentisate 1,2-dioxygenase (EC 1.13.11.5), and 2 protocatechuate 3,4-dioxygenases (EC 1.13.11.3) were revealed. The CYP450 enzymes identified in the genome are likely involved in oxidative transformation of betulin, suggesting their potential role in the bioconversion of this triterpenoid compound.

## Data Availability

This Whole Genome Shotgun project has been deposited in GenBank under the accession number JAJNDE000000000.1, the genome assembly number GCF_021026055.1. Raw sequence reads were deposited in the Sequence Read Archive with accession number SRR17030453 under BioSample accession number SAMN23420023 and BioProject accession number PRJNA783162.
